# How Lifetime Evolution of Parkinson’s Disease Could Shape Clinical Trial Design: A Shared Patient–Clinician Viewpoint

**DOI:** 10.3390/brainsci14040358

**Published:** 2024-04-03

**Authors:** Jules M. Janssen Daalen, Aranka Gerritsen, Gijs Gerritse, Jan Gouman, Hannie Meijerink, Leny E. Rietdijk, Sirwan K. L. Darweesh

**Affiliations:** 1 Radboud University Medical Center, Donders Institute for Brain, Cognition and Behavior, Department of Neurology, Center of Expertise for Parkinson & Movement Disorders, 6525 GA Nijmegen, The Netherlands; jules.m.janssendaalen@radboudumc.nl (J.M.J.D.); aranka.gerritsen@ru.nl (A.G.); 2 Dutch Parkinson’s Patient Association, P.O. Box 46, 3980 CA Bunnik, The Netherlands; gijsgerritse@gmail.com (G.G.); jan.gouman@planet.nl (J.G.); hanniejem@gmail.com (H.M.); lenyrietdijk@gmail.com (L.E.R.)

**Keywords:** Parkinson’s disease, disease-modifying treatment, lifetime research, patient–clinician collaboration

## Abstract

Parkinson’s disease (PD) has a long, heterogeneous, pre-diagnostic phase, during which pathology insidiously accumulates. Increasing evidence suggests that environmental and lifestyle factors in early life contribute to disease risk and progression. Thanks to the extensive study of this pre-diagnostic phase, the first prevention trials of PD are being designed. However, the highly heterogenous evolution of the disease across the life course is not yet sufficiently taken into account. This could hamper clinical trial success in the advent of biological disease definitions. In an interdisciplinary patient–clinician study group, we discussed how an approach that incorporates the lifetime evolution of PD may benefit the design of disease-modifying trials by impacting population, target and outcome selection. We argue that the timepoint of exposure to risk and protective factors plays a critical role in PD subtypes, influencing population selection. In addition, recent developments in differential disease mechanisms, aided by biological disease definitions, could impact optimal treatment targets. Finally, multimodal biomarker panels using this lifetime approach will likely be most sensitive as progression markers for more personalized trials. We believe that the lifetime evolution of PD should be considered in the design of clinical trials, and that such initiatives could benefit from more patient–clinician partnerships.

## 1. Introduction

Parkinson’s disease (PD) is a complex neurodegenerative disorder with ever-increasing incidence [1]. A decades-long stage during which pathology accumulates precedes PD diagnosis. Recent advances in the prodromal phase, specifically the increased number of genetic susceptibility studies and the recognition of prodromal symptoms, have enabled the inclusion of at-risk individuals in early disease courses. Together with the arrival of biological definitions of PD, this creates opportunities for innovative clinical trial designs that acknowledge this lifetime evolution of PD. Specifically, these would include population, target and outcome measure selection. Although hampered by knowledge gaps, a lack of lifetime thinking is exemplified by two newly proposed biological classification and staging systems put forward for staging in PD trials, in which an α-synuclein seeding amplification assay (SAA) is centralized. Briefly, SAA assesses the tendency of aggregation of α-synuclein present in patient material. Although suitable for the detection of α-synuclein aggregation in individuals, SAA is not a reflection of biology. Seeding is a lab-based induction of α-synuclein oligomerization, and some individuals with PD and leucine-rich repeat kinase 2 (LRRK2) mutations have a negative seeding assay. The main outcome of these SAAs is also binary, making them unfit for the quantification of pathology [2]. Importantly, the classification and staging systems do not yet provide a compelling approach to include causal factors that play a role in earlier life. These include measures of neuroinflammation, mitochondrial function and oxidative stress or (exposure to) environmental factors, brain trauma and physical activity throughout disease phases. Due to period effects and effect windows, the timing of such factors has a significant impact on disease course [3]. As such, the current versions of staging systems are inherently limited in their ability to reflect the underlying disease biology over the lifetime course. Therefore, they do not yet provide a framework for the biological stratification of patients for disease trials, nor an approach for personalized outcomes based on causal factors.

Previous studies have suggested including patient–researchers to generate meaningful research scopes, design clinically relevant studies and improve priority setting in research to address these challenges [4,5]. We hypothesized that interdisciplinary collaboration between clinical researchers and patient researchers from diverse backgrounds could generate new research ideas for these challenges in clinical trial design. Therefore, the aim of this study was to discuss the most important challenges and methodological gaps in knowledge about PD. Subsequently, we aimed to develop these gaps into a concept that was shared and supported by all group members. For this reason, we created a workgroup consisting of three researchers and four patient–researchers. In monthly video sessions over a period of 1.5 years, and two-monthly sessions over one additional year, recent research articles were discussed in the light of the concept under-development, namely the lifetime evolution of PD and its consequences for clinical trial design. Patient–researchers took the lead in selecting studies to discuss and provided the studies for participants to read and prepare before the meetings. The clinicians only rarely provided context to scientific debates or hot topics from the field. The professional background and lived experience of the patient–researchers offered a significantly different point of view compared to previous studies. For example, professional backgrounds in causal modelling, fundamental research and veterinary medicine led to discussions about causal interactions, personalized temporal trajectories and protective or risk factors including lifestyle, use of medication and microbiome. We aligned our views into actionable insights that ultimately distilled into the ideas and knowledge gaps presented in this Viewpoint, and that were discussed during multiple sessions in the final year.

Here, we bring forward the impact of a lifetime approach on clinical trial design in this Viewpoint. We define the lifetime evolution approach (‘lifetime approach’) as an approach to design clinical trials that considers the temporal trajectories of the individual exposome, integrates mechanisms relevant to the disease phase with targeting and timing of treatment, and integrates this with pathophysiology-specific biomarkers. As such, it helps to identify knowledge gaps in recently proposed biological definitions of disease, specifically those that are needed to inform future disease-modifying trials on targetable populations, mechanisms and outcomes. As an example, we illustrate how a lifetime approach might influence clinical trial design in two Boxes. As both the patient– researchers and clinicians greatly value the (ongoing) sessions on the current and different topics (e.g., ethical aspects of biomarker research and PD prevention studies), we encourage other researchers to regularly consult–patient researchers as part of their ideas and concept developments.

## 2. The Lifetime Approach on the Individual Level

**Figure 1:**
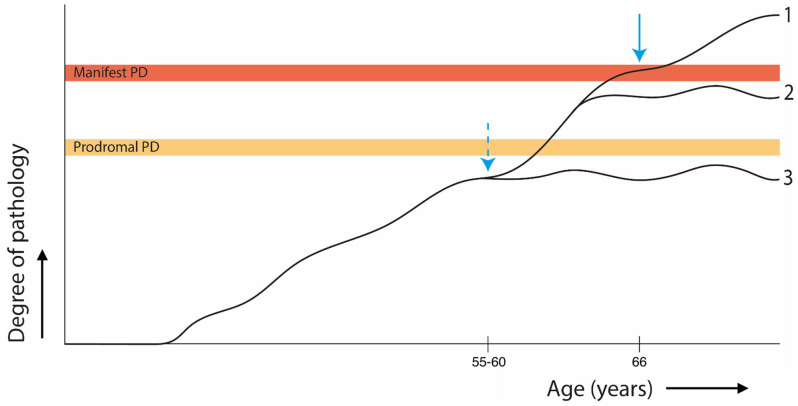
Conceptual model representing the accumulation of Parkinson’s disease (PD) pathology through one (hypothetical) lifetime with diagnosis at age 66, and the putative effect of disease-modifying intervention. 1: Classic development of manifest PD where, early in life, some individuals are exposed to triggers and facilitators of PD pathology. Solid arrow: current age range of de novo study inclusion and mean participation in studies in manifest PD [17,18], where ‘the horse has already left the barn’. 2: Slowing of PD development due to protective factors such as disease-modifying therapies or lifestyle habits. It also represents insufficient presence of facilitators for the development of manifest PD from prodromal PD (gross age ranges depicted for visualization purposes). 3: Individuals who have been exposed to triggers but do not develop sufficient pathology for PD symptoms. Dotted arrow: early intervention opportunities as identified by individual PD risk profile (genetic risk, exposome, positive α-synuclein seeding assay, etc.).

The study group was initiated to address the most basic of questions, namely how PD manifests itself over time. PD has a long, pre-diagnostic phase during which pathological processes accumulate. This phase can be divided into the following three stages: the risk phase, the preclinical phase, and the prodromal phase. Each stage represents different aspects of PD development, from the activation of initial pathophysiological mechanisms to the emergence of early non-motor symptoms [6]. The concept of a lifetime approach for PD research emphasizes the importance of understanding and implementing this pre-diagnostic phase of the disease and the accumulation of pathological processes over a lifetime. A framework for the pathological processes within this pre-diagnostic phase was proposed by dividing factors contributing to PD development into triggers, facilitators and aggravators [3]. According to the concept, every individual will be exposed to different factors at different timepoints. These factors may or may not add to the pathological burden of PD development, depending on the magnitude. Critically, the timepoint of exposure to risk factors in a person’s life plays a crucial role in their future PD development and progression [7]. This has several implications on the individual level. First, every individual is subjected to different exposures to and combinations of triggers, facilitators and aggravators, which leads to a different pathogenesis for each patient (Figure 1). Most unaffected individuals will never be aware of ever having been at an increased PD risk at some point in their lives, having been exposed to triggers with insufficient power (Figure 1, line 3). Exposure effects of triggers, facilitators and aggravators are determined by the critical period, during which exposure to the event is most toxic. This model fits in the chain-of-risk framework of disease, that describes interrelatedness of causal factors. However, it is not known what factors might require each other (A > B > C, i.e., a pure trigger effect), whether risk is accumulative with independent exposure, or whether there is a reinforcing effect of factors on each other. As an example, the pathophysiological role of mitochondrial dysfunction is not disputed, and several PD risk genes are related to different aspects of mitochondrial dysfunction. However, despite being one of the putative most important contributors to PD pathophysiology, temporal trajectories and interactions of mitochondrial function with environmental exposure, physical activity and molecular processes such as lysosomal dysfunction and neuro-inflammation over the lifetime are unknown [8,9]. Furthermore, beneficial factors such as a protective genetic profile [10], increased exercise [11], diabetes treatment [12] or disease-modifying interventions [13] alter the course of pathology development across individuals in early stages. Accordingly, the timing of interventions targeting those factors determines their ultimate efficacy [14,15,16]. Clarifying these relationships will provide better insights into development of neuroprotective or disease-modifying strategies in the early disease course. Also, their potential to enhance reserve, delay decline, or modify the rate of decline in PD on the individual scale can be revealed. On a supra-individual scale, the lifetime approach can have several implications that thereby influence decision-making for clinical trials. In the subsequent sections, we discuss the potential implications for the trial population, interventional target and outcome measures.

## 3. Lifetime Approach and Target Population

After having defined the lifetime approach, we envisioned that by adopting such an approach in PD research, our understanding of pathophysiology would be positively impacted. Accordingly, it would have implications for selecting the target population of clinical trials in the future. If PD risk indeed constitutes an additive effect of multiple risk factors over a lifespan, risk factors or protective factors have a different effect or weight. This does not only depend on the timepoint of exposure, but also on the individual’s mix of protective and risk factors. Furthermore, factors contributing to these processes can have different weights, depending on their interaction with other factors [19]. This is not only the case for factors that add to PD progression, such as mitochondrial dysfunction [20], but also for factors that positively influence the disease course. These are, for example, a protective genetic profile, certain lifestyles or lifestyle interventions and exposure to environmental factors. An increasing number of specific triggers and facilitators are identified and (bio)markers for those factors are becoming available. Consequently, case–control studies can be used to quantify the associated risk and select individuals to inform the relative weight of a factor and its interaction with other factors in pathophysiology. This approach impacts the target population by recognizing early exposure that is already acknowledged in a research setting. For example, previous smoking cessation trials in (young) adults might form an interesting observational or even interventional cohort to investigate the subsequent risk of PD [21]. Such insights can also inform a unique PD roadmap for each patient, as recently proposed in a novel patient-specific pathogenesis model of PD [22]. Examples of where such roadmaps might lead are quantification of the disease-modifying effect of mental stress and environmental toxins such as pesticides. Alternatively, prime examples might be the putative disease-modifying role of the hormonal life cycle and use of contraceptives in women, and the protective effects that this might have during aging [23].

Interdisciplinary research can fill the knowledge gap by taking multiple relevant factors into account when explaining pathologic and phenotypic differences between individuals with PD. Since PD is a multifactorial disorder, identified factors should be jointly investigated [24]. Studies could combine methods such as combining large population studies that investigate pesticide exposure with qualitative evidence of individuals. This can lead to new insights and causal inferences regarding the role of pesticides in PD pathophysiology. This knowledge could be translated into improved population selection by subgroup stratification and, as a consequence, more precise targeted treatment opportunities. On the other hand, population studies could primarily use large research cohorts and revitalize study data to avoid the need to set up of costly and long-duration follow-up studies. This is particularly the case since these risk factors can only be identified and weighted in sufficiently powered cohorts. More accurate identification of protective as well as risk factors will ultimately also facilitate research into the effects of preventive measures for at-risk individuals. Stratifying by or correcting for the (time-dependent) weight of causal and protective factors by subgrouping might decrease unexplained variance. As such, stratification based on disease progression will improve outcome measurement in trials.

Further opportunities exist for multidisciplinary efforts in trial design, as overlap in molecular and pathophysiological pathways between neurological disorders is likely larger than was previously imagined. For example, pathophysiological overlap with other neurodegenerative diseases such as Alzheimer’s disease and genetic ataxias is significant [25,26]. Integrative studies in other neurodegenerative diseases that investigate causal factor interactions, such as the influence of genetic factors and environmental risk factors and pathogens on (auto-)immunological activity can serve as a template for joint research initiatives into PD [27,28]. Although associative studies can point toward important associations, more complex studies are needed to investigate precise time relations and interactions of factors. Studies often disregard this and only look at later stages in life when PD is already in a prodromal or even manifest phase, thereby overlooking this time-dependency. For example, case–control studies that exploit real-world risk factor exposure can give insight in the differential exposure vulnerability in subgroups. If combined with deep phenotyping, this gives the potential to find actual interactive effects of risk factors, appealing common treatment targets, and possibly find different outcomes such as variable PD penetrance, different subtypes or different symptom cascades.

## 4. Lifetime Approach and Treatment Targets

If the lifetime approach impacts PD subtypes and these arise by heterogeneity in involved factors and mechanisms, it might also impact selection of the target treatment for disease modification trials. A better understanding of the sequence of mechanistic events in early pathological stages might give rise to novel treatment targets. The so-called sensitive period of a protective or disease-modifying factor describes the period of optimal effects and has not been determined for many PD-related treatment options, let alone in the context of novel biological subclassification or staging systems in PD. Determining this sensitive period and integrating it into trial design could improve the selection of appropriate study participants and enhance the chances of success for disease-modifying or neuroprotective interventions. Moreover, a recent perspective in this journal illustrates the limitation of PD animal models that do not take into account the temporal complexities of PD on a molecular scale [29]. Despite this, such models are currently the gold standard for compound screening in the preclinical developmental phase. Matching treatment targets to target-specific engagement and outcome markers might be a more pragmatic strategy for future studies to evaluate treatment potency in PD subpopulations.

In various disease-modifying trials, Hoehn and Yahr stages up to 2.5 are eligible, but this might be too late for most compounds to work [13]. For example, antidiabetic glucagon-like peptide-1 (GLP-1) agonists such as liraglutide and exenatide are hypothesized to improve mitochondrial function, inhibit oxidative stress and to induce neuroplasticity and neurotrophic effects [30]. However, potency of induced mechanisms and subsequent impact on the clinical course will steadily decline with delayed administration. The same holds true for the presumed disease-modifying effects of exercise interventions, which will be most efficacious when administered in the prodromal phase to combat chronic neuro-inflammation [11,31]. Of course, the symptomatic and neuroplastic effects and subsequent direct impact on quality life of exercise interventions in manifest PD should not be underestimated [32,33]. Conversely, outcomes of such trials can also give insight into the time-dependent efficacy of such treatments on pathological processes (through intermediate outcome measures) and efficacy (through primary and secondary outcomes). An example is highlighted In Box 1, which shows the microbiome as a potential early target in at-risk populations. As such, earlier detection and improved population stratification will not only enable interventions for secondary or tertiary prevention, but also primary prevention in an at-risk or even broader population, such as a range of lifestyle interventions [11].

Box 1 **The microbiome as an example lifetime approach target or population and outcome stratifier.**

Increasing evidence suggests that microbiome profiles may affect the incidence, age of onset, phenotype and disease course of PD [27,34,35,36,37]. This raises the question of whether microbiome profiles interact with other potential (environmental) risk factors across the life course, which would make the microbiome an appealing target for early life preventive interventions [28]. Studies in other neurodegenerative diseases even suggest the presence of transcriptomic interactions with microbiota in neuronal tissue [38,39]. In addition, the effects of exercise as a disease-modifying intervention might be modulated by its effects on the microbiome [40]. The first exploratory studies targeting the microbiome have been launched [41,42].

## 5. Lifetime Approach and Biomarker Development and Selection

With differing pathological pathways and complex interactive effects between patients, especially across the time domain, the value and specificity of prospective PD biomarkers will likely differ across patients and disease stages. As specific biomarkers and specific PD phenotypes correlate with biological factors, they can be used to identify subgroups from the heterogeneous PD patient group [6,43]. Combined, such biomarkers could be aggregated into biomarker profiles [44]. Aided by the newly proposed PD definitions [45,46], recent initiatives that diligently include well-profiled subgroups in trials and biomarker research form the first step towards stage-specific biomarker profiles [47,48,49].

Temporal associations between biomarker levels and disease progression are only recently being uncovered. For example, with regard to serum biomarkers, inflammatory and anti-oxidant markers such as insulin-like growth factor-1 (IGF-1), urate and leukocyte differential show distinct patterns from the pre-diagnostic into the manifest stage [50]. Furthermore, several interleukins increase with disease severity, non-motor symptom severity and possibly mortality [51,52,53]. Less specific cellular injury-related markers such as neurofilament light-chain increase with disease progression in de novo PD and are associated with motor decline [54,55,56]. On the other hand, neuroplasticity biomarkers can be both imaging and molecular markers, and are likely more valuable in manifest PD [32,57]. Such initiatives are already deployed for the differential value of imaging markers throughout the disease course for both disease state and evaluation of progression [58]. Recently, a multimodal biomarker panel for mitochondrial function, including imaging markers, has demonstrated an association with disease progression in individuals with early PD [59]. With increasing insights into specificity of biomarkers for subgroups over time, the granularity of such overviews will likely increase dramatically.

**Figure 2:**
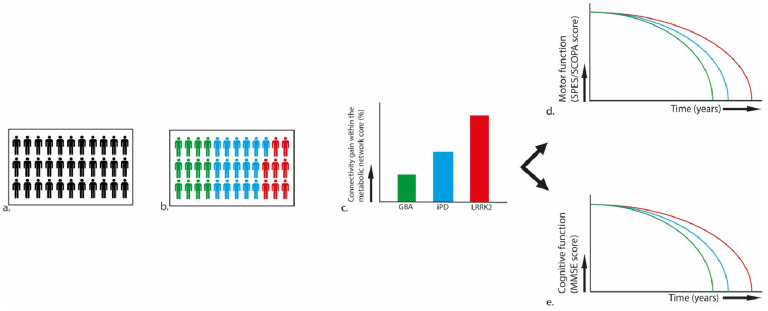
Example of identification of PD subgroups in trial populations based on specific biomarkers and their relationship to specific pathophysiological mechanisms. (**a**) heterogenous PD population. (**b**) subgroups based on specific biomarkers. (**c**) subgroup differences in functional and structural connectivity within the metabolic network core, so called Parkinson’s disease-related patterns (PDRPs). (**d**) subgroup differences in motor function based on Short Parkinson’s Evaluation Scale/Scales for Outcomes in Parkinson’s Disease (SPES/SCOPA). (**e**) subgroup differences in cognitive function based on Mini-Mental State Examination (MMSE). GBA: glucocerebrosidase. LRRK2: leucine-rich repeat kinase 2. iPD: idiopathic PD.

Disease phenotype does not directly result from low-level pathophysiological processes [60], and recent calls have requested the departure from phenotype-based stratification [57]. Still, there might be merit in including phenotypical profiles in such biomarker profiles, with the rise of novel phenotype–pathogenesis correlates, especially in the light of novel PD classification systems. For example, recent evidence for a link between α-synuclein spread and chronology of symptom progression proposes brain-first and body-first subtypes [24]. Furthermore, novel phenotype–genotype association studies link symptom clusters to distinct genetic and inflammatory profiles [61], as is already established for (neuro-)inflammatory markers that distinguish between PD and atypical Parkinsonism [62]. The characterization of early prodromal symptoms, including cognitive dysfunction in individuals with REM-sleep behavior disorder (RBD) 10–15 years before phenoconversion, additionally provides further opportunity for phenotypic markers [63]. Finally, in addition to molecular biomarkers and phenotypical markers, neuroimaging markers can be integrated into such profiles. For example, recent evidence suggests that LRRK2, glucocerebrosidase (GBA) and idiopathic PD (iPD) have distinct Parkinson’s disease-related patterns (PDRPs) in network connectivity, which seem to be independently associated with symptom profile and progression [43,64,65,66]. The integration of such a neuroimaging element in multimodal biomarker profiles is depicted in Figure 2.

The recent boom in omics research in PD is useful to support data-driven biological subtyping to identify biomarkers [67]. If combined with deep phenotyping in populations that are heterogeneous regarding demographic and clinical characteristics, this gives the potential to find actual interactive effects of risk factors that partly explain variable PD penetrance, phenotypes and progression (Box 2) [68]. Hypothesis-generating evidence may yield novel biomarkers in the pre-clinical phase from population-based cohort studies [69]. Such studies can be hypothesis-free or result from the aforementioned observational or case–control studies. Challenges will lie in the heterogeneity of study-specific factors, which hampers aggregation of study findings, and the need for well-matched control groups in population-based studies. Findings could also inform in silico studies of drug development and might thereby ultimately yield novel insight for disease-modifying targets, especially with regard to the potential need for drug cocktails in individuals [68,70]. An example of a next-generation cohort study is the recently launched Cincinnati Cohort Biomarker Program, which is a longitudinal, hypothesis-free investigation focusing on molecular markers and includes people with a broad set of neurodegenerative diseases [68]. Ultimately, by identifying specific biomarkers and biology-defined disease classification and staging, PD will be treated with the use of and monitored by multimodal biomarker panels [6,71]. A recent article provides a first glimpse of how such developments transform the future of clinical trials and patient management [72]. Although the recent proposals for a more biology-based classification and staging system do not yet provide a compelling solution for such stratifications, the increased attention to such systems will likely aid in further developments in this field [46,73]. Future cohort studies could leverage these novel biological staging systems to explore add-ons for stratification, such as mechanistic biomarkers, imaging parameters reflecting biology and wearable technology for motor and non-motor symptoms.

Box 2 **The lifetime approach: biology-specific biomarkers.**

The gold standard MDS-UPDRS part III motor scale is a coarse outcome measure and does not reflect a direct linear relationship between pathological PD markers and disease severity. This is a major drawback for clinical trials taking into account lifetime evolution. Nevertheless, in our discussions, several outcomes that are a better reflection of pathological progression were identified, including PD-related pattern (PDRP) [74] and specific imaging properties (e.g., substantia nigra free water content on MRI). These outcomes are included in the newly proposed definitions of PD [45,46] and, importantly, are associated with specific phenotypes [74,75,76].

## 6. Ethical considerations and Limitations

The lifetime approach for clinical trials is currently a mostly conceptual model, as biological definitions and ways of stratifying potential trial participants are in their infancy, limiting the number of variables. Furthermore, limited knowledge about the sensitive period of treatments and preclinical models that use too simplified versions of PD on which such treatments are assessed hamper translation to clinical trials. This means going back to the drawing board to reconsider the limitations of preclinical models, and focusing on developing treatment-specific target engagement markers to evaluate their potency in different PD subpopulations. Another important limitation is the lack of knowledge about sensitivity of most putative disease biomarkers to disease progression, which is essential to perform accurate power calculations. Longitudinal biomarker studies, ideally within existing cohorts, will fill in such disease profiles step by step and increase the granularity of population–treatment matching. Several other more practical challenges exist. The impact on quality of life following the disclosure of an individual’s risk profile for PD is only beginning to be explored [77]. At the same time, being eligible for disease-modifying trials as a result of a specific risk profile opens up possibilities for contributing to valuable causes, potentially improving well-being. Unavoidably, implementing such a lifetime approach in clinical trials touches on several privacy-related issues for trial participants. More detailed knowledge on private and occupational life are necessary to allow for improved participant selection. In addition, this approach might complicate the inclusion of trial participants, significantly increase trial costs and increase the burden of trial participation, as several screening steps, including analysis of several markers, might be necessary in order to assess participant eligibility. Furthermore, this approach can complicate statistical modeling, as several additional covariates have to be considered.

## 7. Conclusions

Through a patient–clinician working group collaboration, we conceptualized the lifetime approach to Parkinson’s disease and envisioned its impact on population, treatment target and outcome selection in clinical trial design. A lifetime approach in PD research influences clinical trial design as it accounts for the cumulative effect of risk and protective factors in the target population, the timing of interventions, and the selection and integration of biomarkers. Although this is by no means a comprehensive overview, we believe that the lifetime approach could contribute to a better understanding of knowledge gaps in PD pathogenesis, as well as clinical trial design. The lifetime approach essentially involves the integration of a chain-of-risk model, as discussed, with a biological staging system that trials may use to select populations, targets and outcomes from. Thereby, it ultimately homogenizes the selection of study participants and improves population–treatment matching, at the same time enhancing the chances of success for disease-modifying interventions. Understanding critical windows and sensitive periods impacts treatment target selection. As temporal associations between biomarker levels and disease progression are being uncovered, specific biomarkers can be indicative of different disease stages and phenotypes. Integrating molecular biomarkers, phenotypical markers and neuroimaging markers into comprehensive biomarker profiles can enhance our ability to characterize PD and evaluate disease progression. As such, multidimensional stage-dependent biomarker panels might assist in accelerating the development of personalized drug trials and, ultimately, more personalized disease-modifying treatment. Specifically, existing longitudinal cohorts to study biomarker sensitivity are warranted. This viewpoint illustrates how patients and clinician–researchers can work together as partners to identify priorities for research on PD. This could inspire other researchers across the field of PD and beyond to use a participatory health research approach.

## Data Availability

No new data were created or analyzed in this study. Data sharing is not applicable to this article.
